# Precision radiation therapy in the modern era of multidisciplinary care in oncology: What matters to our patients and beyond?

**DOI:** 10.1016/j.jncc.2024.05.004

**Published:** 2024-05-11

**Authors:** Feng-Ming (Spring) Kong

**Affiliations:** The University of Hong Kong- Shenzhen Hospital, Li Ka Shing Faculty of Medicine, Hong Kong, China

The current standard of cancer care is combined multimodality therapy, including surgery, radiation therapy, chemotherapy, target therapy, and immune therapy. Radiation therapy, as one of these five major cancer modalities, has a significant contribution to the cure of cancer patients. According to the 2006 data of the Surveillance, Epidemiology, and End Results (SEER) Program, an estimated 3.05 million cancer survivors received radiation therapy, accounting for 29% of all 5-year cancer survivors.^1^ The number of radiation-treated cancer survivors is projected to 4.17 million by 2030. The radiation-treated female survivors were at 58% and projected to increase to 66% in 2030.^1^ It was estimated that about 50% of cancer patients in developed countries such as Australia indicated for radiation therapy,^2^^,^^3^ while less than 10% patients in low-income countries had access to such a life-saving treatment.^4^^,^^5^ Interestingly, the availability of radiation therapy has direct correlation with the survival of the cancer patients^6^ or the mortality to incidence ratio (MIR) of cancer.^7^ The high-income countries have lower MIRs than many low/middle income countries where over 50% and 90% of patients requiring radiotherapy do not have access to this life saving treatment.^7^ While the reason of such discrepancy is multifactorial, limited knowledge on the role of radiation therapy is an important reason. Investing in radiation therapy was associated with more life-saving and economic benefit, as presented in a special issue in *Lancet Oncology*.^8^ This highlights the importance of promoting the data and evidence in radiation therapy, justifying this very first special issue of the *Journal of the National Cancer Center* (*JNCC*) to promote the research and clinical activity on the role of radiation therapy.

Radiation therapy, with a 120-year's history of success in cancer treatment, is evolving in indication and technology, along with the advances in target therapy and immunotherapy and extension of the life expectancy of cancer patients. Modern radiation therapy can now be quite conformal to the level of “targeting” local treatment modality that is consistently improving with the state-of-the-art technology. In this very first special issue of *JNCC*, the readers will learn the role of radiotherapy, particularly in its integration with the advanced systemic therapy in **this modern era of multidisciplinary cancer care**, with precision oncology, intelligent oncology, and immune oncology. The role of local radiotherapy needs to be personalized and individualized with combined consideration of the systemic drug and specific stage of the cancer and the patient.

**In the era of precision oncology**, precision approaches of radiation therapy have already been applied throughout the treatment procedures and have achieved steady improvement in precision during the process. Beginning from superficial radiotherapy over a hundred years ago, radiation technology has advanced from 2-dimentional (2D), 3D Conformal Radiation Therapy, 4DRT, imaging guided radiation therapy (IGRT), to intensity-modulated radiation therapy (IMRT) and volumatic arc treatment. Such precision approach of radiation therapy has been highlighted with the implementation of stereotactic body radiaton therapy (SBRT) or stereotactic ablative technology (SABR) which delivers ablative doses "targeting" the cancer while allowing for safe protection of normal tissues and has already generated outstanding results across various types of cancers. From this special issue of *JNCC*, the readers will learn what matters to our patients in the treatment outcomes of various types of conformal therapy in a wide range of cancers.

The guideline and evidence for the role of radiation therapy in lung cancer, the top cancer killer, is summarized by the investigators from the Netherlands in the review titled “Radiotherapy for small cell lung cancer in current clinical practice guidelines” (https://www.sciencedirect.com/science/article/pii/S2667005422000059). The long-term outcomes of post-chemotherapy consolidation radiotherapy are reported for Asia patients with extensive stage small-cell lung cancer in “Feasibility and long-term outcomes of post-chemotherapy-based consolidation radiotherapy in extensive stage small-cell lung cancer” (https://www.sciencedirect.com/science/article/pii/S2667005423000431). In addition to validating the potential benefit demonstrated from patients in Western countries,^9^ this study further confirmed the role of thoracic consolidation radiation therapy and small field precision technique which reduced in-field recurrences without increasing out-of-field recurrences or Grade-5 toxicity. Meanwhile, the readers will learn the significance of radiation induced esophagitis in survivors of non-small cell lung cancer (NSCLC) at https://www.sciencedirect.com/science/article/pii/S2667005421000077. This study for the first time comprehensively evaluated the impact of radiation esophagitis with conditional survival assessment and demonstrated that this side effect, though itself not life threatening, is associated with inferior survival in NSCLC patients treated with radiation therapy, suggesting the importance of precision radiation therapy.

**Fig. 1:**
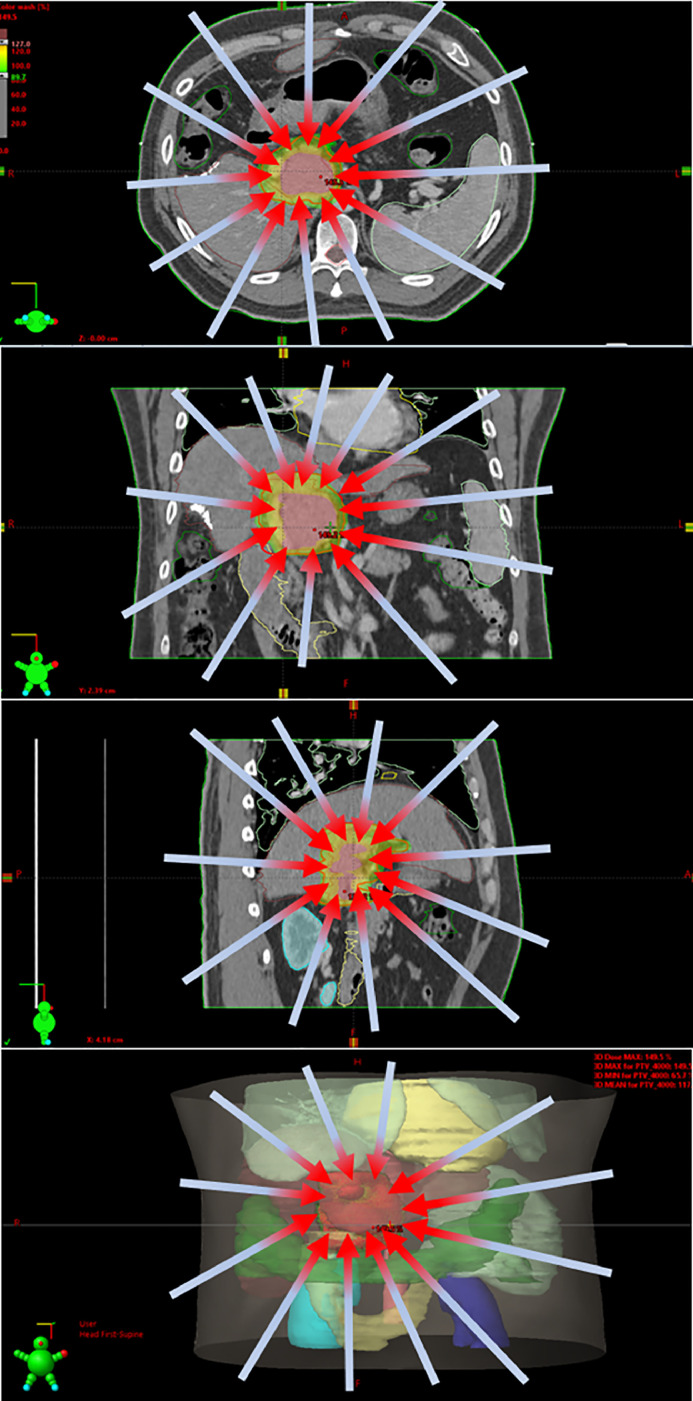
Schematic illustration of dose map for stereotactic body radiation therapy (SBRT) "targeting" a high dose to the tumor (Red in the middle) while minimally radiating the adjacent normal tissues in a patient with a recurrent hepatocellular carcinoma. Red color in the front of the arrows shows high doses of radiation, while light blue indicates remarkably lower doses.

Radiation therapy plays an increasingly important role in treating primary and metastatic liver cancers. Historically, radiation was primarily used for palliation due to concerns about radiation-induced liver diseases. With advances in radiation technologies, such as IMRT, SBRT, sophisticated motion management, abdominal compression, respiratory gating, and 3D imaging from CT scans that allow for precise targeting of cancerous tissues while minimizing damage to healthy tissues, modern conformal radiation therapy has generated outstanding treatment outcomes in liver malignancies. Chinese investigators performed a comprehensive review and summarized the role of high precision SBRT (Fig. 1 shows an example plan) in hepatocellular carcinoma in guidelines and evidences from the East and the West (https://www.sciencedirect.com/science/article/pii/S2667005422000333). They reviewed the evidence and guideline adoptions of SBRT, and compared the results with other local modalities including surgery and the differences in SBRT adoption between guidelines from eastern and western countries. For metastatic liver cancer, the readers will learn from randomized trials conducted from North America like SABR-COMET and STAMPEDEF the evolving role of radiation therapy in various stages of the disease, from oligometastatic to widely spread metastatic liver cancers (https://www.sciencedirect.com/science/article/pii/S2667005422000400).

Treatment outcomes and associated factors of precision radiation for various other cancers are also presented in this special issue. Precision-based tailored radiotherapy is applied in the multidisciplinary management of pediatric central nervous system tumors (https://www.sciencedirect.com/science/article/pii/S2667005423000248).

Quality of life and survival outcomes are reported for patients with inoperable esophageal squamous cell carcinoma after definitive radiation therapy in a multicenter retrospective observational study in China from 2015 to 2016 (https://www.sciencedirect.com/science/article/pii/S2667005423000261). The results of radiation therapy in combination with other modalities, other than radiation factors, like the time interval between surgery and postoperative radiotherapy, are presented for major salivary gland carcinoma (https://www.sciencedirect.com/science/article/pii/S2667005422000345). Axillary lymph node dissection plus radiotherapy that improved overall survival in pTxN3 occult breast cancer patients than those receiving chemotherapy, may be an optimal strategy for those patients (https://www.sciencedirect.com/science/article/pii/S2667005422000631). Personalized precision radiotherapy and its evolving role for human papillomavirus-positive oropharyngeal cancer are also presented (https://www.sciencedirect.com/science/article/pii/S2667005422000953).

Quality and safety assurance procedures, one of the essential components in precision radiation therapy, are exampled through a multicenter protocol on hippocampus avoidance in whole brain radiation from Europe through the article titled “Quality assurance and safety of hippocampal avoidance prophylactic cranial irradiation in the multicenter randomized phase III trial (NCT01780675)” (https://www.sciencedirect.com/science/article/pii/S2667005423000297). Although this study had a focus on procedures and events of protocol violations were not powered for conclusion, numerically more failures were noted in underdosed areas, suggesting the importance of the quality insurance on RT. More extensive future works are needed.

**In the era of intelligent medicine,** radiation oncology needs to be intelligent as well. Intelligent oncology is a fast-growing field that has already started to integrate oncology, radiology, pathology, molecular biology, multi-omics, and computer sciences to promote cancer prevention, screening, early diagnosis, and precision treatment. Advanced artificial intelligence (AI) technologies such as natural language processing, machine/deep learning, supervised or unsupervised learning, computer vision, and robotic process automation, have been increasingly used in radiation oncology. AI is expected to play a pivotal role in all fields of oncology, including clinical patient care, teaching, and clinical research. Indeed, during the era of intelligent oncology, radiation therapy can be “intelligentized” to be more precise, faster, and more cost-effective during every step of the radiation procedures, from decision making, simulation, target delineation, treatment and quality assurance planning, delivery, and outcome follow-up.^10^ This special issue provides a grand introduction to intelligent oncology through https://www.sciencedirect.com/science/article/pii/S2667005422000928, followed by an overview of artificial intelligence in radiation oncology and medical physics (https://www.sciencedirect.com/science/article/pii/S2667005423000479), and a review on automated organs at risk and target volume contouring for precision radiation therapy in the modern era (https://www.sciencedirect.com/science/article/pii/S2667005422000655).

The host immune system is important for cancer killing through radiation and radiation can reshape the immune status of the patient.^11^ **In this era of immunotherapy,**the effect of radiation immune modulation cannot be over emphasized. While the immune stimulation effect of radiation has been tested in numerous clinical trials, the immune suppressive effect of radiation, though long known, has been largely neglected. Here we present a holistic view of radiation-immune interaction through the review article “Prospect of radiotherapy technology development in the era of immunotherapy” (https://www.sciencedirect.com/science/article/pii/S2667005422000175). **Immune preserving radiation technologies are needed**, along with future research on immune stimulating dose regimen, spatially fractionated RT (SFRT), proton therapy, and ultra-high dose rate irradiation (also known as FLASH).

Finally, **precision radiotherapy in this modern era of multimodality care needs to integrate biomarkers** available in clinic for dynamic monitoring. A study that revealed dynamic tumor mutational burden change (∆bTMB) in combination with residual circular tumor DNA (ctDNA) improves survival prediction for locally advanced NSCLC patients with chemoradiotherapy and consolidation immunotherapy (https://www.sciencedirect.com/science/article/pii/S2667005424000218), highlighted the significance of such a personalized approach in detecting molecular residual diseases, assessing treatment response, and guiding personalized immunotherapy in that patient group. For patients with residual ctDNA, consolidation immunotherapy provided greater benefit in overall survival and progression-free survival. Dynamic ∆bTMB was predictive of treatment outcome. The combined model integrating post-chemoradiotherapy ctDNA and its changes as a biomarker has the potential to improve predictive accuracy for survival and tumor progression. Clearly, biomarkers or models with comprehensive consideration of tumor, patient, treatment, and societal factors are needed to advance precision oncology to a new horizon of success.

I sincerely hope the readers will enjoy the learning from this special issue of *JNCC*. Importantly, I believe the knowledge and skills on radiation oncology provides a most cost-effective strategy of life extension and/or quality improvement.
